# Recurrent Pericarditis in a Woman With Acromegaly Responding Favorably to Transsphenoidal Surgery

**DOI:** 10.1016/j.aace.2025.01.002

**Published:** 2025-01-11

**Authors:** Samantha Jacobson, Jonathan-Raphaël Stetco, Natasha Garfield

**Affiliations:** 1 Faculty of Medicine, University of Sherbrooke, Sherbrooke, Quebec, Canada; 2 Division of Endocrinology, McGill University Health Centre, Montreal, Quebec, Canada

**Keywords:** acromegaly, pericarditis, growth hormone, insulin-like growth factor-1, cardiovascular complications

## Abstract

**Objective:**

Acromegaly is associated with increased insulin-like growth factor 1 (IGF-1), promoting systemic inflammation and cardiovascular complications. We present a patient with acromegaly who developed recurrent pericarditis, resolving soon after somatotroph pituitary adenoma resection. The objective of this report is to describe a case of uncontrolled acromegaly with recurrent, unexplained pericarditis.

**Case Report:**

A 46-year-old woman was referred after a neurologist identified a 9 mm pituitary lesion on magnetic resonance imaging. Laboratory tests showed elevated IGF-1 of 52.3 nmol/mL (12.3–32.9 nmol/L), a nonsuppressible growth hormone (GH) level of 3.8 mcg/L (<0.4 mcg/L) after a 75 g oral glucose tolerance test, confirming acromegaly. One-year postdiagnosis, the patient developed pleuritic chest pain from pericarditis with moderate-to-severe pericardial effusion. Symptoms resolved with nonsteroidal antiinflammatory drugs, colchicine and pericardiocentesis. Over 3 years she experienced multiple episodes of recurrent pericarditis. A comprehensive diagnostic workup, including rheumatologic and infectious evaluations, was negative. After transsphenoidal adenoma resection, IGF-1 normalized, and medical therapy was discontinued. Pericarditis recurred 2 months postoperatively but has not occurred again over 12 years of acromegaly remission.

**Discussion:**

Hypersecretion of GH in acromegaly leads to elevated IGF-1 levels, which affect inflammatory responses. IGF-1 can promote systemic inflammation through proinflammatory cytokines, its effects may vary depending on tissue type. In this case, resolution of pericarditis following IGF-1 normalization suggests that elevated IGF-1 levels may mediate the inflammatory process in the pericardium.

**Conclusion:**

The case suggests that acromegaly may predispose some patients to pericarditis, but its frequency and underlying pathogenesis remain unclear.

**Highlights**

• Recognize pericarditis as a potential complication in acromegaly patients
• Elevated IGF 1 levels may trigger inflammatory mechanisms leading to pericarditis
• This case highlights the importance of looking and monitoring for cardiovascular health in acromegaly patients

**Clinical Relevance**

This case highlights the importance of monitoring and managing cardiovascular complications in acromegaly, emphasizing the need for early intervention and hormone control to prevent serious conditions like recurrent pericarditis.

## Introduction

Acromegaly is a rare endocrine disorder caused by excessive growth hormone (GH) secretion, with a prevalence ranging from 2.8 to 13.7 per 100 000 people.^1^ It typically results from a GH-secreting pituitary adenoma, leading to elevated insulin-like growth factor 1 (IGF-1) levels.^2^ Clinical manifestations include enlarged facial features, hands and feet, alongside systemic complications, including cardiovascular diseases like left ventricular hypertrophy and cardiomyopathy.^2^^,^^3^ Arterial hypertension and valvular heart disease are common, with electrocardiographic abnormalities frequently observed.^3^

Although cardiovascular complications are recognized in acromegaly, pericardial abnormalities are exceedingly rare. To date, only a single case of chronic pericardial effusion in a patient with active acromegaly has been reported, with elevated IGF-1 levels.^4^ The occurrence of recurrent pericarditis in patients with acromegaly has not been previously described in the literature.

Here, we describe a 46-year-old patient who experienced multiple episodes of pericarditis during hormonally active acromegaly. This case highlights the temporal association between active acromegaly, recurrent pericarditis, and resolution of episodes following definitive treatment and normalization of IGF-1 levels, suggesting a potential causal relationship.

## Case Report

A 46-year-old woman was referred to our clinic after a neurologist, in the evaluation of headaches, identified a 9 mm lesion in the right hemianterior pituitary gland on brain magnetic resonance imaging (MRI). The patient complained of worsening headaches over several months, described as 'pressure-like pain'. Her medical history was positive for an oophorectomy for an ovarian cyst decades earlier, a history of cholelithiasis requiring cholecystectomy, and recurrent urinary tract infections. Her only chronic condition was thalassemia minor. She was not taking any medications other than over-the-counter analgesics for headaches and reported no prior cardiovascular disease. On physical examination, her visual fields were intact to confrontation. She was noted to have an enlarged nose and increase nasolabial folds. Her blood pressure was 110/65 mmHg and no signs of cardiac murmurs, joint swelling, prognathism, enlarged tongue, hoarse voice, or enlarged hands and feet. Laboratory testing revealed IGF-1 of 52.3 nmol/mL (12.3–32.9 nmol/mL) and a nonsuppressible GH of 3.8 mcg/L, following a 75 g oral glucose tolerance test (<0.4 mcg/L). Prolactin (16.8 ug/L; reference range of 3.3-26.7 ug/L), AM cortisol (301 nmol/L; reference range of 120-535 nmol/L), adrenocorticotropic hormone (7 pmol/L; reference range of ≤ 10 pmol/L), thyroid-stimulating hormone (1.35 mIU/L; reference range of 0.4-4.40 mIU/L), free T3 (3.9 pmol/L; reference range of 3.5-5.7 pmol/L), luteinizing hormone (2.6 IUslLiter), follicle-stimulating hormone (7.7 IUslLiter), and estradiol (174 pmol/L) were within normal limits.

The patient was diagnosed with acromegaly, and somatostatin analog therapy was initiated with octreotide, which initially decreased her IGF-1 to 35.1 nmol/L (12.3–32.9 nmol/mL). On follow-up 8 months later, her IGF-1 subsequently rose to 52.1 nmol/L (12.3–32.9 nmol/mL), prompting a switch to lanreotide.

Approximately 1 year after diagnosis, the patient developed pleuritic chest pain. Electrocardiogram revealed diffuse ST elevations with T wave inversions and transthoracic echocardiogram showed moderate-to-severe pericardial effusion secondary to pericarditis with no structural heart anomaly. No etiological workup was done. Pericardiocentesis was performed and she was treated with nonsteroidal antiinflammatory drugs (NSAIDs), colchicine, leading to symptom resolution. Two years later, she had a second episode of pericarditis. She was treated again with NSAIDs and colchicine, which resulted in symptom resolution. Her IGF-1 level was 40.6 nmol/mL on lanreotide (12.3–32.9 nmol/mL). Over the next year, the patient experienced three additional episodes of pericarditis. Each episode was managed with a combination of naproxen, colchicine, and prednisone. During one episode, she developed a pericardial effusion, which required pericardiocentesis. Echocardiography revealed a left ventricular ejection fraction of 57%. Tests including ANA, anti-SSA, anti-dsDNA, c-ANCA, p-ANCA, C3, C4, and ACE levels, were noncontributory. Tests for syphilis, tuberculosis, and HIV, were negative. Laboratory results showed elevated C reactive protein at 150 mg/L (reference range of 0–5 mg/L) and microcytic anemia (hemoglobin: 102 g/L; reference range of 120–160 g/L, mean cell volume: 65.2 fL; reference range of 82.0–100.0 fL). Her liver function tests (bilirubin total: 18.0 μmol/L; reference range of 1.7–18.9 μmol/L, alanine aminotransferase: 16 U/L; reference range of 6–45 U/L, alkaline phosphatase: 71 U/L; reference range of 42–98 U/L), renal function (creatinine 50 umol/L; reference range of 40-85 umol/L), thyroid-stimulating hormone (0.92 mIU/L; reference range of 0.4-4.40 mIU/L), troponin-I (0.05 ug/L; reference range of 0.00-0.06 ug/L), and creatine kinase (61 U/L (reference range of 5-140 U/L) were all within normal limits. She exhibited no symptoms of arthritis, uveitis, or cutaneous manifestations of vasculitis. Her IGF-1 level at that time was elevated at 82.8 nmol/mL (11.4–31.1 nmol/mL). The patient was thus referred to neurosurgery for pituitary lesion removal.

**Fig. 1:**
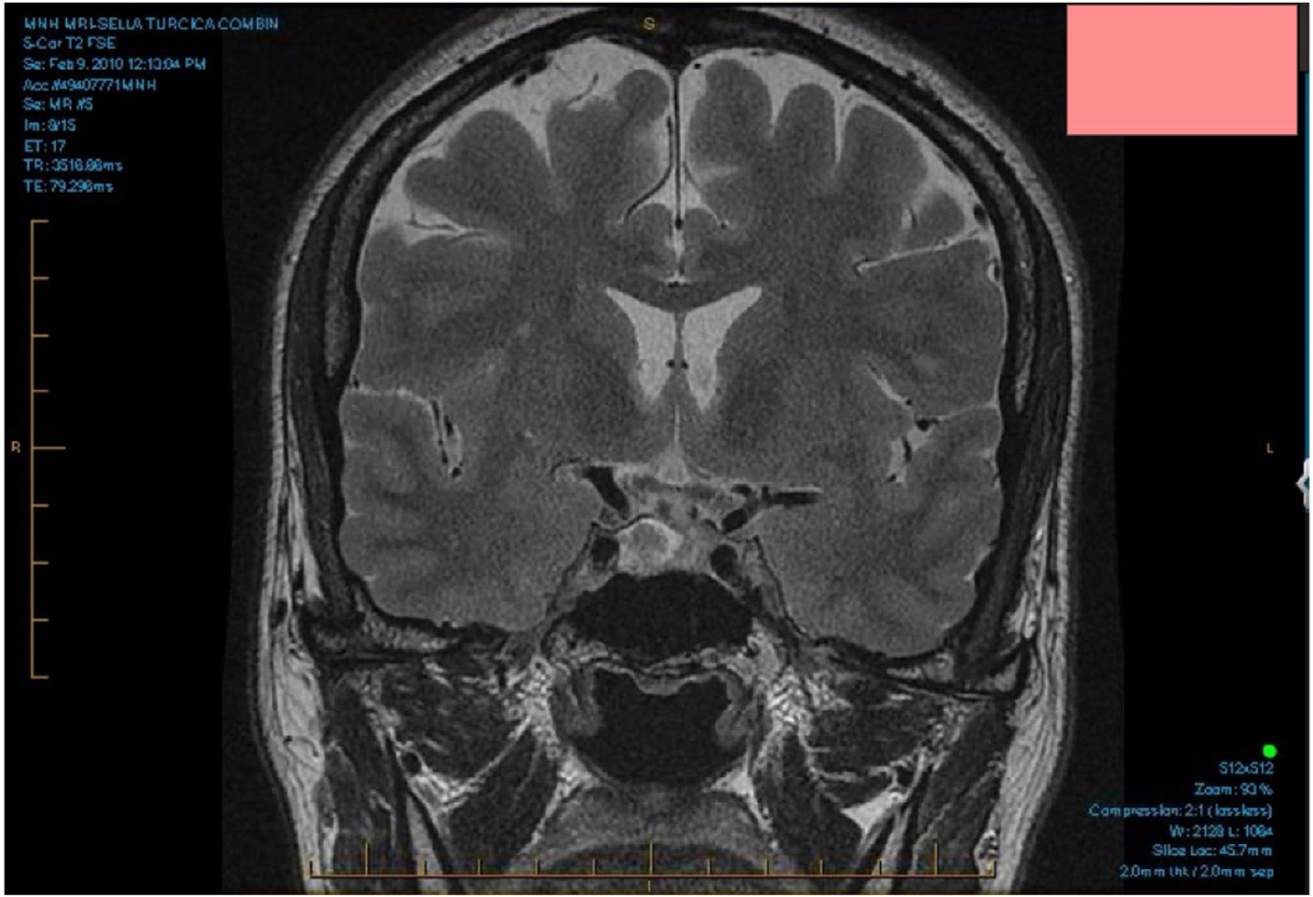
Preoperative brain magnetic resonance imaging (MRI); Showing a 1.8 cm pituitary adenoma in right hemianterior pituitary gland.

Preoperative brain MRI revealed a 1.8 cm lesion in the right hemianterior pituitary gland, compatible with a pituitary adenoma (Fig. 1). Nine months following her previous pericardial episode, she underwent a transsphenoidal resection of the pituitary adenoma. Histopathology of the resected adenoma confirmed a somatotroph pituitary adenoma. Postoperatively, her IGF-1 dropped to 31.2 nmol/mL (11.4–31.1 nmol/mL), and she stopped all her medications, including lanreotide, colchicine, and naproxen.

**Fig. 2:**
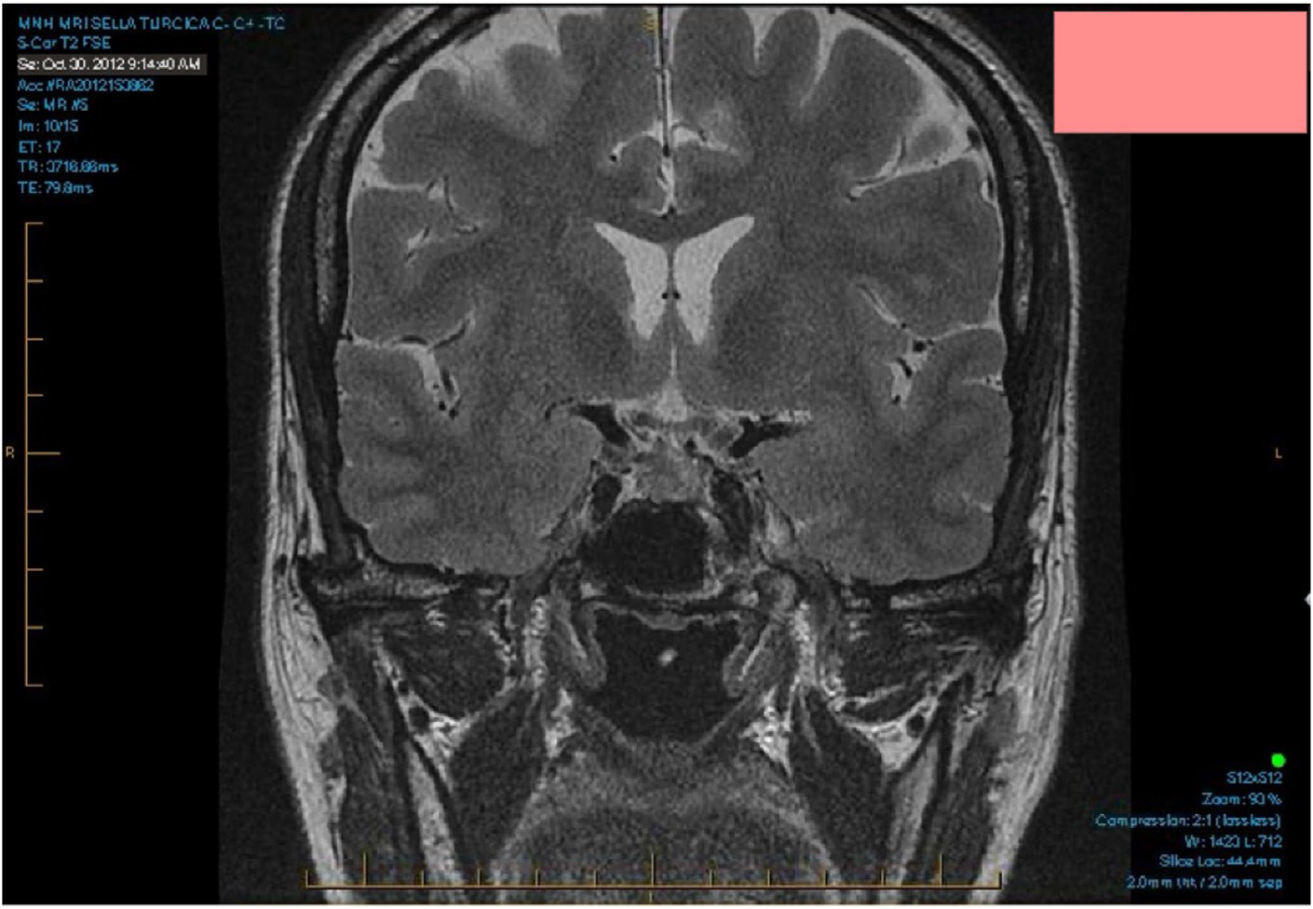
Brain magnetic resonance imaging (MRI) post-transsphenoidal pituitary adenoma resection; Showing no evidence of recurrence of the pituitary adenoma.

Two months after surgery, despite an improvement in IGF-1 at 29.6 nmol/mL (11.4–31.1 nmol/mL) and GH to 0.32 mcg/L (0.03–4.00 mcg/L), the patient experienced a final episode of pericarditis. This episode was treated with NSAIDs and colchicine, leading to full resolution. One-year postoperative brain MRI findings were compatible with resection of right-sided pituitary adenoma, with no evidence of recurrence of the lesion previously operated (Fig. 2).

**Fig. 3:**
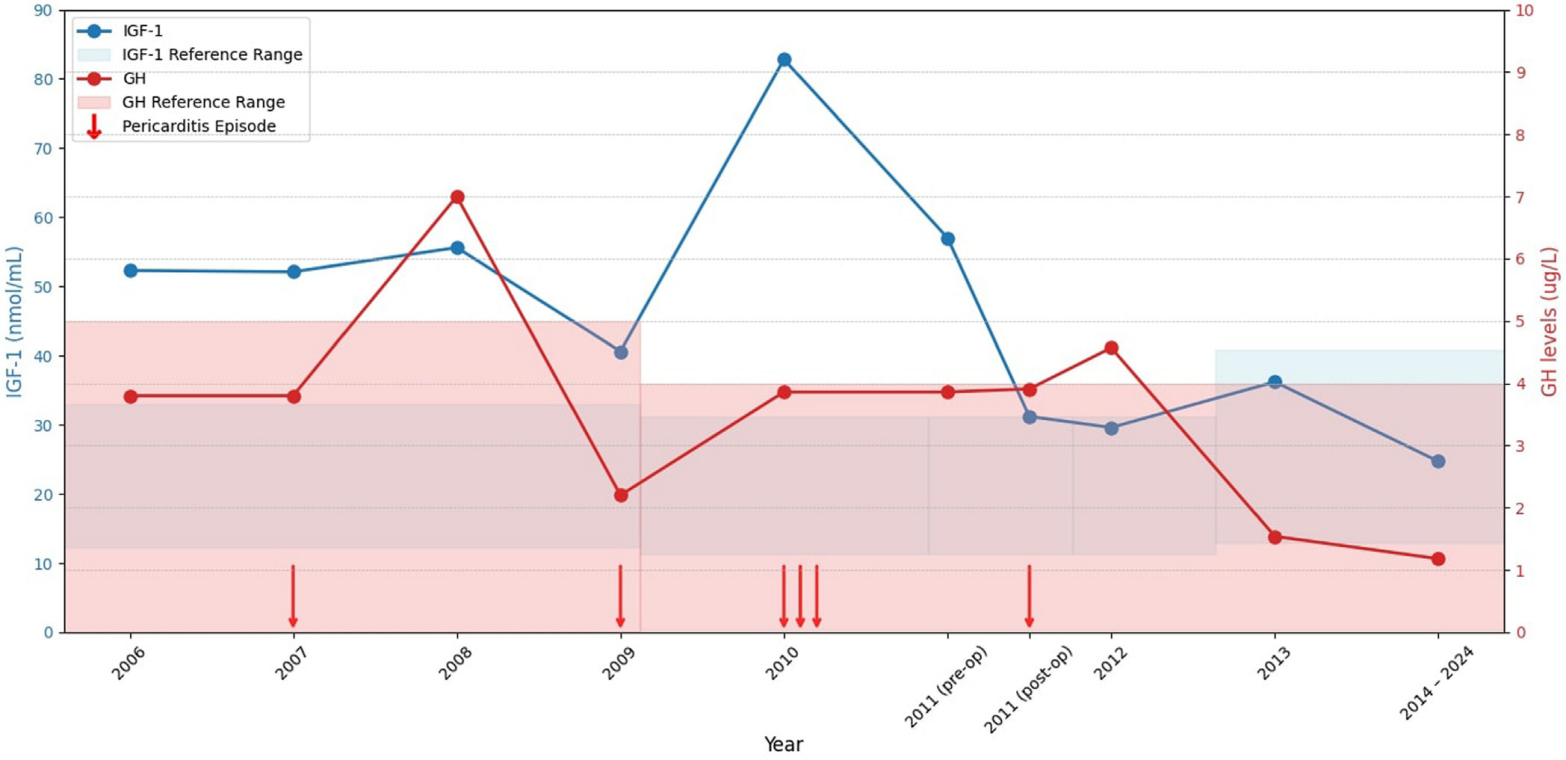
Trends of IGF-1 and GH levels with pericarditis episodes (2006-2024), with reference ranges highlighted. Red arrows indicate the timing of pericarditis episodes. *GH* = growth hormone; *IGF-1* = insulin-like growth factor 1.

Since her pituitary surgery, the patient has been followed-up for a period of 12 years. During this time, the patient has not experienced any recurrent episodes of pericarditis. Her acromegaly remains in remission with an IGF-1 of 10.7 nmol/L (7.3–22.1 nmol/L). No adjuvant therapy was required. There were no other complications of acromegaly including hypertension, colonic polyps, diabetes, change in ring and shoe size, or arthralgias. As of her most recent visit in 2024, she has continued to report no symptoms of recurrence, including chest pain or headaches. Fig. 3 summarizes the trends in IGF-1 and GH levels over time and their association with her acute pericarditis episodes.

## Discussion

We present a case describing a 46-year-old woman with acromegaly who experienced six episodes of pericarditis over several years. The majority of these episodes occurred while her acromegaly was active. Acromegaly is characterized by excessive secretion of GH and IGF-1, leading to systemic complications, including cardiovascular abnormalities. Common manifestations include left ventricular hypertrophy which can lead to heart failure and cardiomyopathy^3^ and arterial hypertension which is an independent risk factor for mortality in acromegaly.^3^ Valvular heart disease often involves the aortic and mitral valves and can persist even after treatment.^3^ Pericarditis, to our knowledge, has not been described in the literature as a feature of acromegaly, although a case of chronic pericardial effusion in a 73-year-old woman with active acromegaly has been documented.^4^ The authors of that report suggested that the effusion was likely linked to pericardial disturbances associated with elevated GH and IGF-1 levels.^4^ In our case, the recurrent nature of the pericarditis and its resolution with IGF-1 normalization further support a possible causal relationship. The pathophysiology of pericarditis in acromegaly may involve inflammatory and immunologic mechanisms influenced by elevated GH and IGF-1 levels.^5^^,^^6^

The episodic flares of pericarditis appeared to correlate with increased IGF-1 levels. To note, IGF-1 was only measured during endocrinology follow-ups, which occurred after her initial cardiology hospitalizations for pericarditis. As shown in Fig. 3, her highest IGF-1 level (82.8 nmol/L) coincided with her most severe episodes of pericarditis. This temporal relationship underscores the potential influence of active acromegaly on her condition. IGF-1 has been shown to potentiate the production of pro-inflammatory cytokines (interleukin 6, tumor necrosis factor alpha, and interferon-γ) in response to toll-like receptor ligands.^6^ These proinflammatory cytokines play critical roles in inflammatory diseases.^7^ Therefore, elevated IGF-1 could upregulate systemic inflammation, potentially triggering or exacerbating episodes of pericarditis.

Most common causes of pericarditis, including autoimmune disorders or infections,^8^ were ruled out in this patient. Additionally, she did not take medications known to cause drug-induced pericarditis, such as procainamide, hydralazine, methyldopa, isoniazid, or phenytoin.^8^ The absence of alternative etiologies strengthens the plausibility of this association. The final episode occurred postoperatively, despite a normal IGF-1 (29.6 nmol/mL), and resolved with reinitiation of colchicine and NSAIDs. This episode followed the abrupt discontinuation of anti-inflammatory therapy, a known risk factor for pericarditis recurrence.^9^ Recurrent pericarditis develops in 15% to 30% of cases.^10^ Risk factors for recurrence include early corticosteroid use, lack of response to NSAIDs, and elevated C reactive protein levels.^11^

At the time of her diagnosis of acromegaly, medical management was the preferred alternative to surgical treatment.^12^ Therefore, a decision was made to treat her medically to shrink the tumor, with the goal of making surgery a viable curative option in the future. As such, she was initially treated with octreotide LAR, which was discontinued and switched to lanreotide after her IGF-1 levels failed to decrease adequately.

While this case adds to the limited understanding of cardiac complications in acromegaly, it is observational and lacks mechanistic validation. Future research should explore the underlying mechanisms linking acromegaly and recurrent pericarditis, as well as the role of medical therapy in preventing recurrence.

## Disclosure

The authors have no conflicts of interest to disclose.
